# Social and ethical implications of data and technology use on farms: a qualitative study of Swedish dairy and pig farmers

**DOI:** 10.3389/fvets.2023.1171107

**Published:** 2023-08-22

**Authors:** Charlotte Doidge, Jenny Frössling, Fernanda C. Dórea, Anna Ordell, Gema Vidal, Jasmeet Kaler

**Affiliations:** ^1^School of Veterinary Medicine and Science, University of Nottingham, Nottingham, United Kingdom; ^2^Department of Disease Control and Epidemiology, National Veterinary Institute (SVA), Uppsala, Sweden; ^3^Department of Animal Environment and Health, Swedish University of Agricultural Sciences, Uppsala, Sweden

**Keywords:** responsible innovation, precision livestock farming, power, identity, focus groups, pig farmers, dairy farmers

## Abstract

**Introduction:**

Livestock farmers are being increasingly encouraged to adopt digital health technologies on their farms. Digital innovations may have unintended consequences, but there tends to be a pro-innovation bias in previous literature. This has led to a movement towards “responsible innovation,” an approach that questions the social and ethical challenges of research and innovation. This paper explores the social and ethical issues of data and technologies on Swedish dairy and pig farms from a critical perspective.

**Methods:**

Six focus groups were conducted with thirteen dairy and thirteen pig farmers. The data were analysed using reflexive thematic analysis and a digital critical health lens, which focuses on concepts of identity and power.

**Results and discussion:**

The analysis generated four themes: extending the self, sense of agency, quantifying animals, and managing human labour. The findings suggest that technologies can change and form the identities of farmers, their workers, and animals by increasing the visibility of behaviours and bodies through data collection. Technologies can also facilitate techniques of power such as conforming to norms, hierarchical surveillance, and segregation of populations based on data. There were many contradictions in the way that technology was used on farms which suggests that farmers cannot be dichotomised into those who are opposed to and those that support adoption of technologies. Emotions and morality played an important role in the way animals were managed and technologies were used by farmers. Thus, when developing innovations, we need to consider users’ feelings and attachments towards the technologies. Technologies have different impacts on farmers and farm workers which suggests that we need to ensure that we understand the perspectives of multiple user groups when developing innovations, including those that might be least empowered.

## Introduction

In Sweden, like many other European countries, the number of farms is decreasing but the size of farms is increasing, particularly for the two most common species, pigs and cattle (1, 2). Appropriate herd health management is required to ensure these farms are sustainable (3). Technologies have been shown to improve health and productivity on farms and literature on the development of precision livestock farming implies that it can help farmers make better decisions (4). Furthermore, farm technologies are often framed positively in media and policy documents (4, 5). Consequently, there is a bias towards pro-innovation in the literature, where an increase in the uptake of technologies is sought (6) and livestock farmers are being increasingly encouraged to adopt digital health technologies on their farms (7).

Swedish dairy farms are generally thought to have a high degree of technology use (7). For example, Swedish dairy farmers have one of the highest proportions of adoption of automatic milking systems worldwide (8). Other technologies that may be used include automatic feeders and sensors that detect activity, oestrus, lameness, and rumination time. Swedish farmers’ experiences of using technologies for adult dairy cattle has been investigated (9, 10), but less is known about their experiences with technologies for youngstock. Numerous technologies have been developed for pig farming such as sensors that measure drinking behaviour (11) and pen level activity monitoring (12). Yet there has been very little research into the uptake of technologies on pig farms (13). Currently little is known about the state of technology use on Swedish pig farms, although it is suggested that many farmers use herd monitoring software technology called PigWin (14).

In the human health field, the discipline of critical digital health studies has emerged in response to the uncritical consideration of technologies (15). This involves an examination of the wider social, cultural, ethical, and political roles that technologies play in peoples’ lives. Studies also seek to understand how technologies change how bodies are understood, visualised, and managed (16, 17). Critical digital health studies takes perspectives from multiple theoretical backgrounds (15). Rich and Miah (18) argue that digital technologies should be approached both from a biopolitical and embodiment perspective to understand the broader complexities of their implications. A biopolitical perspective is particularly useful for studying the power relations between digital health technologies and the users (17, 19, 20). Whereas, an embodiment perspective investigates users selfhood and identity (21, 22).

Most literature related to livestock technologies do not consider their social and ethical contexts (23). However, agricultural social scientists have started to approach digital farm technologies through a more critical perspective. Whereas digital critical health scholars focus on the relationship between people and technologies, agriculture has the added complexity of including the relationship with livestock animals. Digital innovations may have unintended consequences and these consequences may vary for different people and animals. This has led to a movement towards “responsible innovation,” an approach that questions the social and ethical challenges of research and innovation (24, 25). This approach suggests that innovators should be responsive to these social and ethical challenges through an interactive process with stakeholders (26). Therefore, relevant stakeholders should have input throughout the technological innovation process (24). A user-centred design approach ensures that the needs and decision-making practices of farmers are considered, as well as the ethical, legal, and social acceptability of an innovation (25, 27). These considerations are often missed in the development of digital farm technologies (23).

Co-creation and user-centred approaches also help to anticipate and address potential negative or positive consequences of the technology (28). Not all experiences can be dichotomised into consistently positive and consistently negative. Therefore, there is a move towards “ambivalence” as an approach to evaluate the experiences of digital health technologies (29–31). Something can be conceptualised as both positive and negative and may depend on factors such a social history, social situations, relationships and organisational or cultural environments (31).

This study was part of a larger EU-funded project called DECIDE, which is described in greater detail in the methods. The project-intends to develop technologies to aid Swedish dairy and pig farmers to control and manage diseases on their farms through a user-centred design approach. Based on the goals of responsible innovation, there was a need to anticipate the potential consequences of technologies on these farms so that we could design technologies in a way which would reduce negative impacts and build upon positive impacts. Therefore, this paper explores the social and ethical issues of data and technologies related to animal health on Swedish dairy and pig farmers’ management practices from a critical perspective. To do so, we make use of theoretical concepts from digital critical health studies, which are further elaborated in the following section, to investigate the social, cultural, ethical, and political roles of technologies on farms. We draw on focus groups with dairy and pig farmers to understand their experiences, perceptions, and feelings towards technologies.

### Theoretical background—embodiment and biopower

The field of critical digital health studies uses the concept of human embodiment to understand how digital data is used to portray the body. Technologies such as social media can allow people to construct their self-identities by posting selected images and descriptions for followers to see (32, 33). Another method of digital embodiment is through the use of digital tracking technologies which aim to make unseen parts of the body (e.g., heart rate, step count, sleeping pattern) more visible (34, 35). Technologies can change the way bodies are understood and experienced. As a result, Lupton (36) suggests that technologies can be seen as extensions of the body to form a “digitised cyborg assemblage” (37).

In agriculture, precision livestock technologies collect continuous data on animals to produce digital representations of the animal (11). Such detailed digital representations have been termed digital doubles (25). It has been suggested that precision livestock technologies generates attitudes that treat or turn animals into objects in which the embodied experiences of and relationships with animals are lost (38). Technologies are not neutral within the human-animal relationship as they affect how animals are perceived and treated (39). These human-animal-technology relations have mainly been investigated for automatic milking systems technology (40–43). Findings suggest that the introduction of automatic milking systems requires that both farmers and cows learn to use the robot via forms of embodied learning (9). The robot also requires training and adaptation by farmers and cows to function sufficiently (40, 44). Representations of a good cow changes to include acceptance of the robot and a body shape that conforms to standards that comply with the robot (42, 44). In contrast, there is little investigation into human-animal-technology relations with respect to youngstock. It has been suggested that calves are marginalized on dairy farms because of their low perceived value compared with adult cows and a lack of integration into the dairy farm system (45, 46). Lack of technologies available for youngstock and lack of data monitoring may play a role in this marginalization because farmers are unable to measure calf performance (46). The marginalization of youngstock suggests that farmers relations with youngstock may be different from adult cows.

Farm technologies may also change farmers’ identities and how they represent themselves. Farming culture has a set of values and standards which farmers keep to, to gain social standing and an identity as a good farmer (47). Symbols of this good farming identity include having meaningful relationships with their livestock and having “hands on”—embodied—knowledge of the livestock (41, 48, 49). Some precision livestock technologies can reduce the need for farmers to have close, personal relationships with their animals and change their roles on the farm. This can prevent uptake of technologies as these values do not align with their good farming identities (50). Furthermore, technologies may erode the traditional farmer identity of being a good stockperson (50, 51). Instead, new farmer identities as data observers, gatherers, and validators may form (52).

Digital critical health scholars also draw on Foucauldian theory, in particular his concepts of governmentality and biopower (53). Foucault’s notion of “biopower” encompasses power in population and individual forms. At the individual level, biopower comprises of disciplinary power and focuses on the subjectification of the body (54). Disciplinary power allows people to govern themselves based on feelings of being observed (hierarchical surveillance) and comparing to norms (55). At the population level, biopower comprises of regulatory power, which includes collecting data of populations (e.g., births, deaths) to generate knowledge of that population. These two power levels allow biopolitical (e.g., life and health) interventions by authorities to work (56). Digital health technologies can enhance the capacity of both disciplinary and regulatory biopower (17).

Although Foucault failed to consider human-animal relations in his analysis of power, critical animal health scholars have developed his work to conceptualize this (57). Research suggests that biopower is relevant to livestock as farmers control the life of livestock populations through management practices such as selective breeding and biosecurity (58, 59). In terms of technology use, Holloway (60) uses the concept of biopower to understand how automatic milking systems produce bovine subjectivities by disciplining the cows’ bodies to conform to technological requirements.

Closely related is the concept of governmentality, which is the way in which a population is governed to maintain and improve the condition of a population (53). Techniques of government are used by authorities to attempt to regulate human conduct (61). One example of a technique of government is the gradual collection of statistical data from farms in Britain (62). This allowed the state to intervene in farming practices by generating policies based on the statistical knowledge that was accrued. Digital technologies on farms also represent a technique of government as they produce new knowledge. Agritech firms, for example, have used this knowledge to shift farming identities around what a good farmer should do and what they should know (63).

Both Foucauldian approaches and embodiment approaches are therefore relevant to investigating technology use on farms. We aim to use these two approaches to analyse Swedish pig and dairy farmers’ perspectives on technology use and their social and ethical implications. We have included youngstock management in our analysis of dairy farms as there is a paucity of information at this age group.

## Methods

### Case and study context

The case we draw on is part of the DECIDE project,^1^ which is a 6 years project funded by the EU Horizon 2020 programme (64). The aim of the project is to develop data-driven tools to aid the control of non-EU regulated diseases in pigs, poultry, cattle, and salmon. The project consortium involves people with expertise in veterinary epidemiology and diagnostics, data science, mechanistic and predictive modelling, economics, animal welfare and social sciences. In addition to academic institutions, the consortium includes a knowledge exchange organisation, laboratory, and technology company. This study focuses on two Swedish cases: tools for dairy and pig farmers. These cases are following a “Living Lab” approach. Some of the key features of Living Labs include co-creation with potential users, using real life environments, using multiple methods, and involving different types of stakeholders (65, 66). This project uses the FormIT Living Lab methodology, which is made up of three iterative stages: concept design, prototype design and innovation design (67). Each stage also has three phases: appreciate opportunities, design, and evaluate. The paper centres on the appreciate opportunities phase of the concept design stage which aimed to obtain a rich picture of farmers, their behaviours, attitudes and values. This stage occurred before any concept or prototype for a tool was developed. Thus, this enables us to anticipate potential social or ethical consequences of potential innovations.

To provide some context of Swedish farms, there are around 2,795 dairy farms in Sweden with an average herd size of 106 cows (68, 69). Sweden was early to adopt a systematic disease prevention strategy through national control and eradication programmes. As a result, dairy herds in Sweden are free from many diseases which are endemic in other countries, including infectious bovine rhinotracheitis, bovine viral diarrhoea, tuberculosis and paratuberculosis (69, 70).

There are approximately 1,175 pig herds in Sweden, of which 61% keep sows for breeding and 80% rear pigs for fattening (68).The average herd size for breeding herds and fattening herds was 175 sows and 951 pigs to market weight, respectively. In similarity with dairy herds, the control strategies mean that Swedish pig herds are free from diseases such as porcine epidemic diarrhoea, and porcine reproductive and respiratory syndrome (70).

### Study design

We used focus groups as the data collection method for this study because we wanted to explore technology and data from farmers’ own perspectives and understand their experiences. The group interaction within focus groups helps to uncover participants’ experiences and perspectives through a process of “sharing and comparing” (71). We conducted the research within a critical realist paradigm. That is, we acknowledge that “the experience of reality is different for different people in different contexts” (72). The focus groups provide us with the farmers’ perception of their reality. We chose critical realism as it allows us to consider how social structures and the material world (e.g., technologies) shape farmers’ perspectives.

### Ethics

The study was approved by the ETH Zurich Ethics Committee (application: EK 2021-N-224). Participants provided written, informed consent by completing a form.

### Researcher characteristics and reflexivity

The researchers had an active role in generating the knowledge for this study. All the researchers involved were female.

CD is a postdoctoral researcher in the veterinary social sciences. She helped to design the discussion guide, analysed the transcripts, and wrote the manuscript. She does not have a strong farming background and is not a vet but does have an understanding of livestock farming from her PhD work. She is a researcher from the UK, and is therefore positioned an “outsider” for Swedish farmers (73).

JF is a veterinary epidemiologist with long experience from research on and implementation of animal health surveillance. She has been educated in social science research methodology for use in veterinary epidemiologic research and has been involved in previous focus group studies in Sweden. She translated and adjusted the discussion guide, and was the moderator of the cattle focus group.

FD is a veterinary epidemiologist and expert in animal health surveillance and digitalization. She is familiar with some of the main animal health data sources used by Swedish farmers and has conducted previous discussion groups with advisors and vets about animal production data.

AO recruited the participants and contributed to the translation of the discussion guide. She was the assisting moderator in the cattle farmer focus group and later the moderator of the pig focus groups. She is a veterinarian with several years of experience from working as a farm health advisor and practitioner.

GV is a veterinary epidemiologist and post-doc researcher working with animal health data from Swedish farms. She has several years of experience from working as a farm health advisor and practitioner in different countries. She has received education in social science methodology for epidemiologic research and has been involved in previous social studies in agricultural communities.

JK conceptualised the study, was involved in designing the discussion guide, supervising analysis, reviewing, and editing the original draft of the manuscript. JK is a veterinary epidemiologist from UK with extensive research experience in use of social science methods for decision-making research.

### Sampling approach

We used a pragmatic approach to sampling based on the information power concept (74). We required a diverse sample to cover our broad study aim. We believed that the focus group method would provide us with good quality dialogue because of the group interaction, the quality of the discussion guide, and the skills of the researchers conducting the focus groups. Additionally, we wanted to explore perspectives across two different livestock sectors (dairy and pigs). An experienced qualitative researcher was conducting the analysis. Finally, we also considered the costs of transcribing and translating the focus group recordings. Based on these factors, we thought that 2–4 focus groups per species would provide us with sufficient information power. We inspected the richness of the data after three focus groups per species and from this we determined that we had enough information to answer our study aim.

A convenience sample was obtained by authors contacting farmers within their network. The author who contacted farmers was a practicing veterinarian and therefore knew some of the characteristics of farmers when inviting them to participate. Eighteen cattle farmers were contacted and only one declined. Among pig farmers, 28 were contacted and nine declined. The reasons for declining were not being able to participate at the suggested dates or not having the time to participate in general. Six alternative dates and time points were suggested to both cattle farmers and pig farmers. The participants were offered 700 SEK compensation for the time they spent in the focus groups.

To allow for a diversity of participants, the only inclusion criteria were that the farms needed to be based in Sweden. For example, we did not exclude participants based on farm size, level of technology use, gender, or role. We aimed to include participants who used technologies on their farm and those that did not. Focus groups with pig and dairy farmers were conducted separately. A total of six focus groups were completed with 26 participants (Table 1). There were 16 female participants and 10 male participants. The ages of participants ranged from 20 to 63 years. The herd size of the dairy farms ranged from 95–470 cows. The herd size of the pig farms ranged from 80–1,100 sows. This means the study sample reflected participants with a diversity of herd sizes. However, we did not have any participants with dairy herd sizes which were much lower than average (106 cows). The pig farm sample included fully integrated and semi-integrated farms. One pig farm and two dairy farms were organic production systems.

**Table 1:** Participants of the focus groups.

Farm type	Focus group number	No. of participants	Male/female	Age range
Dairy	1	4	0/4	25–48
	2	5	2/3	36–50
	3	4	2/2	34–50
Pig	1	5	2/3	25–58
	2	4	2/2	20–63
	3	4	2/2	27–62

### Data collection

Online focus groups were held using Microsoft Teams. Online focus groups were chosen because we could include participants who were geographically dispersed. A topic guide was used to facilitate the discussions. The topic guide was developed by authors who were experienced in qualitative methods (CD and JK). Following the Living Lab approach, the guide used an appreciative inquiry technique which included some positively framed and future oriented questions (75). The questions were first tested over the phone with two farmers in the UK. Then, two pilot focus groups were held in the UK. After the first pilot, we decided to change the order of questions so that the future-oriented questions were placed at the beginning of the discussion. We felt that this improved the flow and breadth of discussion in the second pilot. The topic guide included the topics of goals for the future of their farm, data use, technology use, and disease management and is included in the Supplementary material. The dairy focus groups were directed towards the management of youngstock, but the pig focus groups were not directed towards a specific age range. Each focus group lasted around 1 h 30 min.

The focus group discussions were held between March 8th, 2022 and May 31st, 2022. The pig focus groups were moderated by AO and assisted by FD. The dairy focus groups were moderated by JF and assisted by AO. There were no other people present in the focus groups apart from the moderator, assistant, and participants. At the start of the sessions, the moderator introduced themselves and their role, the aims of the DECIDE project and the aims of the focus group. The assistant moderators took notes during the focus groups. All focus groups were audio and video recorded with the participants’ consent. The recordings were transcribed verbatim by an independent company. The focus groups were held in Swedish language and were translated into English to allow for analysis by non-Swedish speakers (CD and JK). The translations were conducted by the same independent company as the transcription. Each translation was reviewed by a second translator at the company and then checked by a Swedish-speaking author (JF, AO, and FD). The English language versions were read by CD who added comments to the document where she needed clarification from authors who were familiar with the Swedish farming context. The Swedish-speaking authors had a timestamped copy of the Swedish language transcription of the focus groups and the English language translation. This allowed the authors to refer to the original recordings if there were any concerns with the translated version. The Swedish speaking authors made edits to the translations, added comments to provide context and answered any questions the UK authors had. These edited documents were then used for analysis.

### Data analysis

Data were analysed using reflexive thematic analysis (76). We chose this analysis method because we aimed to generate patterns of shared meaning across the dataset. CD led the initial analysis. Transcripts were read multiple times and noting down preliminary ideas. Initial codes were generated inductively from the data which was facilitated by NVivo. The initial codes were mostly based on semantic meaning at this stage. The transcripts were further coded with a critical digital health lens, focusing on embodiment and Foucauldian concepts. Codes were grouped together to form initial themes, which reflected patterns of shared meaning. These were then revised by checking consistency with the data and through critical discussions with JK. We presented the revised themes in a meeting with JF, FD, and GV to receive their perspective. Based on their feedback, the themes were revised one final time.

## Analysis and discussion

The themes generated from the analysis are summarised in Table 2.

**Table 2:** Summary of the themes.

Theme	Description
Extending the self	Farmers’ concepts of their self-identity extend to the way they manage their animals and the data they produce
Sense of agency	Farmers value their experiential knowledge, affective relations with their animals, and their ability for abductive reasoning
Quantifying animals	Technologies generate new quantitative knowledge about animals’ behaviour which forms new norms that animals adhere to at the individual, group, and herd level
Managing human labour	Technology that collects data on farm workers and animals is used as a surveillance technique to manage the workers. It can also facilitate collaboration between people on the farm

### Extending the self

This theme centres on how farmers’ self-identity is shaped by their animals and how farming norms form methods of self-regulation. Low morbidity and mortality on farms were uncritically accepted as the ideal situation for farms. It had become an embedded norm in both pig and dairy farming.

“The goal is always to have zero deaths and to have zero diarrhoea and zero pneumonia.” Speaker 3, dairy focus group 3.

“The way you want it in the future is never having to see a diarrhoea, for example in a calf, and things like that. It would be an incredible relief to avoid it.” Speaker 1, dairy focus group 3.

This low morbidity, low mortality ideal had become a moral imperative. Farmers appeared to put responsibility on themselves to work towards this ideal. Legislation also fostered the farmers position of care for their animals. Achieving this ideal was seen as an indication of hard work and dedication. Thus, farmers took part in a form of moral self-regulation. They carry out a moral assessment of the mortality/morbidity levels on their farms and choose their own boundaries of what constitutes good levels of mortality/morbidity. Jaye et al. (77) suggest that livestock act as moral capital because they require care of the farmer to flourish. Low mortality is one way of demonstrating moral capital.

“Our goal is to have 0% calf mortality and we have gotten there, but it has been a lot of work so far.” Speaker 3, dairy focus group 2.

Farmers took part in “body projects” to construct their self-identity in relation to the norms around low mortality. Human beings often work on the body as a project of their identity. Some examples include using digital self-tracking devices to monitor diet and exercise (17) or reproduction (78) and express feminine identities. However, our analysis shows that the animals were seen as an extension of the farmers’ bodies. For example, the quotes below show that the health status of their animals can make farmers feel positive or negative emotions. Farmers’ sense of identity emerges from their thoughts and feelings on the effects of interactions with their animals. Thus, the animals can be seen as an extension of the self and become a target for purposeful action. Shilling (79) name this bodily and emotional unison between animals and humans a “companionate” inter-species body pedagogics. Their description of companionate body pedagogics centres on people’s bonds with companion animals. We suggest that this can be broadened to encompass farmer-farm animal bonds.

“Then you have had one that has passed away, which almost never happens, but if there is one, you get sad and then you work even harder next time.” Speaker 1, dairy focus group 2.

“We then took the step to build on our own piglets, and for that we would move to the best health status in Sweden. So, today we run (a large, specialist) production. It is totally different. Now it is a joy to never find a pig coughing in the slaughterhouse.” Speaker 3, pig focus group 2.

Examining data can act as a technique for self-improvement. In many of the quotes, farmers referred to herd-level data such as the number or percentage of deaths in their herd. They used this data as an opportunity to reflect on their performance. When farmers experienced the death of an animal, they reflected on their management and attempted to improve it. This may also trigger farmers to collect more data. For example, the quote from (Speaker 2) below shows how she started collecting data to see if a change in colostrum practices would improve her performance. Thus, farmers use technologies of the self to manage their animal care practices. Technologies of the self are what Foucault conceptualised as “specific techniques that human beings use to understand themselves.”

“Now, maybe two or three die each weaning. It might not be a lot on 700 pigs, but we do not like it.” Speaker 4, pig focus group 2.

“It will be very interesting to follow and see. Before we gave them frozen colostrum, but it has lost a lot when you thaw it, I think, so now we have started giving them fresh milk instead. We will see what happens, but so far, the calves have reacted very positively.” Speaker 2, dairy focus group 1.

Technologies of the self are not just restricted to numerical data. They can also include written texts such as diaries, letters or social media posts (80). One example given by Speaker 1 and Speaker 4 is the use of a notebook to write down any changes made in farm management.

“I listened to a podcast, and I thought it was such a great tip that you should have a notepad where you write down all the changes you make. Just the date and then the kind of feed you use and when you start etc. We also check the brix values. It’s difficult to remember everything. What caused this change? I thought that it was a great idea to write everything down.” Speaker 1, dairy focus group 1.

“I had maybe six dead the first time but after that I was very tough on myself, so I wrote down every little thing I did every day and I changed and adjusted every time I weaned, so now I feel like we have a really good routine for this.” Speaker 4, pig focus group 3.

The farmers may also share their data with other people such as their vet. Thus, the farmers invite vets to monitor their data as a self-management technique. The gaze of veterinarians and their regulatory bodies may induce farmers to perform self-surveillance.

“Yes, in my case anyway, they (vet) always check to see if I have been good and registered and organized.” Speaker 4, pig focus group 1.

In summary this theme shows how norms around animal health shape farmers’ identity. Farmers project their identity through managing their animals. They do so by using self-management techniques which include inspecting their animals’ data and documenting changes in their practices.

### Sense of agency

This theme focuses on the value of farmers’ subjectivity and agency, i.e., the ability to act on their own volition. Many farmers talked about how they hold tacit knowledge about their animals. This was often expressed as “djuröga” in Swedish (having an eye for the animals, i.e., an ability to see and understand how the animals feel and what they need). Animals showed signs and symptoms of being ill such as not eating as quickly as usual or standing away from their group. These signs cannot always be fully conveyed in words. This experiential knowledge has been studied in multiple contexts including sustainable farming (81) and antibiotic use (82, 83).

“I do not usually look at much, I prefer to look at the animals. I am out here all the time anyway. You know more about your animals when you see them every day than if you only check on them now and then.” Speaker 4, dairy focus group 1.

“Yes, an eye for animals. The calf drinks a little. Deviant behaviour as well. There are certain behaviours that you can use to detect diseases early on. You can see the disease the day before it is on its way, and it is very important to tackle it. It’s about an eye for animals to learn to see the problem.” Speaker 5, dairy focus group 2.

We draw attention to the bodily and affective sensations that form an important part of farmers’ experiential knowledge, an aspect which is often missed in previous research contexts. Some signs and symptoms of disease can only be experienced through sensations and actions. For example, Speaker 1 places her hand on her pigs to feel and understand their health status. This shows how farmers’ bodies and animals’ bodies are interconnected via sensations and generate affective responses.

“I probably just feel the sows most of the time. When you have been doing this for 30 years, or over 20 years you get a bit like—you just put your hand on them and say, “You have a fever, now I know, good".” Speaker 1, pig focus group 3.

In another example of affective responses, (Speaker 3) talked about how her employee uses her olfactory senses to identify when their pigs will become ill. This employee was able to draw on her “olfactive frames of remembering” to understand what constitutes a socially acceptable, or socially unacceptable, scent identity for the pigs (84). This scent identity then affects how she manages the pigs.

“But (employee) who works with us, she can smell when they are bad. She just says, “Here, they are bad,” and then you know that if you do not treat them directly, it will be really bad the next day. She has a sensibility for that… She just says, "Here it is", and then you just treat. It is never wrong. If we do not treat, then they are bad the next day. She gets it right.” Speaker 3, pig focus group 2.

Some of the farmers thought that technology could not do the same job as their own knowledge and experience. Indeed, it is common discourse that technologies facilitate farmers’ decision-making rather than substituting them in the decision-making process (4, 85). The farmers thought that technology cannot experience the animals in the same way because technologies cannot engage in affective or sensory processes in the way that human and animal bodies do. Yet, technologies can be affective and sensory in a different manner (22). For example, a thermometer can give an indication of the body temperature of an animal, which facilitates the farmers’ sensory knowledge of the animal. However, technologies such as robots may remove this human body-animal body sensory relation.

“I think it’s difficult to integrate technology because it depends on your eye for the animals. All of you here know this. Certain things you need to do by hand when pigs are being born and such. It’s hard to have a robot for this.” Speaker 4, pig focus group 3.

“Yes, there it is difficult with AI stuff, to just feel or smell; I do not know if computers can handle that yet.” Speaker 3, pig focus group 2.

“I was a little worried when we got the robot. With a pen or tied up or however you milk by yourself, then you see all the cows and touch all the cows twice a day, but with a robot you lose that contact. I would never have a robot without measuring rumination, or I would totally lose control.” Speaker 1, dairy focus group 1.

One way in which technologies had affective relations with farmers was through giving farmers feelings of control. For example, farmers sometimes mentioned how they felt out of control if technology misfunctioned because they may be unable to fix it. On the other hand, technologies may also help farmers to feel in control of situations which need a level of detail or where they cannot be present in-person.

“We have time-controlled feeding and that can be messy if the technology isn’t working properly.” Speaker 3, pig farmer focus group 3.

“We want to get them into an automatic milk feeder eventually to distribute the meals and to have better control as well. Now we are checking with buckets how much they eat but I am guessing that an automatic milk feeder can warn in another way.” Speaker 2, dairy focus group 3.

Farmers were able to use a mixture of data and their own reasoning to experiment with their management practices. The pig farmers, in particular, often discussed how they have had to stop using zinc oxide due to the 2022 ban from the European Commission on its oral administration to food producing species (86). As a result, farmers had to figure out other practices to reduce diarrhoea in piglets, and many discussed the adoption of a process of “tinkering.” This requirement for abductive reasoning, mixing intuition and reasoning, is a human skill which is difficult for technologies to emulate. This is because abduction requires creativity in which seemingly unrelated concepts are explored and connected (87, 88). Thus, when new challenges emerge on farms, technology may facilitate finding a solution, but this still requires the agency of the farmer to make new connections between patterns and make new decisions. The generation and integration of new knowledge by farmers through experimentation and talking to others (e.g., vets and farmers) is important for the adaptive capacity of the farms (89).

“We also stopped using zinc, maybe a year and a half ago, and so far one pig has died. Now we do not actually have that many animals, but perhaps over 5,000 piglets anyway; one has died, and a total of maybe 50 treatments during this time. So we have succeeded fantastically well.” Speaker 3, pig focus group 2.

“Here it comes down to the eye, and to somehow combine both what we have written down but also figure it out with a veterinarian, to know how to do. So it is all about the personal decision making in the situation, and try things out, because there are no clear directions here on what to do. So far I think that one tries to tinker a lot with these things oneself. What is the right feed and what is not.” Speaker 5, pig focus group 1.

This theme therefore shows how farmers value their own agency to manage their farm animals. Farmers have abilities in abductive reasoning, creativity and human body-animal body sensory relations which cannot be replaced by farm technologies. However, technologies may be able to facilitate these processes.

### Quantifying animals: individuals, groups, and populations

#### Individuals

This theme centres on how farmers’ conceptualisations of their animals are shaped by their data. Although in the previous theme we suggested that animals’ data is an extension of the farmers’ self (i.e., identity), the animals’ data also shapes how the animal is viewed by the farmer. Indeed, Gabriels and Coeckelbergh (80) argues that “concepts of self and other are co-created at the same time as being reshaped by them.”

Many of the farms were embedded in technologies which make the bodies of pigs and dairy cows more visible. For example, technologies could detect animals’ movements and their eating and drinking habits. When thinking about what makes a useful technology, farmers often talked about technologies that could measure and identify problems that were not visible to the human eye.

“It should be something that could find something quicker than the eye.” Speaker 7, pig focus group 1.

“When you look at the calves, what is it that you can see? It’s whether they have stomach aches or diarrhoea. You can often see that for yourself. But if you could see the heart rate, if you could see whether the heart rate is increased or the respiration. Especially with lung issues, if you could catch it faster. That would be great.” Speaker 3, dairy focus group 1.

Technologies were used to alert farmers of deviances in animals’ eating and drinking behaviours. This was often at the individual animal level. Animal behaviours therefore became quantified and generated new knowledge about what constituted normal behaviour for individual animals. Farmers were able to use this new knowledge to target animals which did not fall into normal standards. It also generated new conceptualisations of what a healthy animal was and new parameters to be measured against. Thus, data on feeding and drinking behaviour became “medicalised.” For example, Speaker 5 (pig focus group 2) uses changes in water consumption to identify diarrhoea in their pigs.

“When we wean, it gives an alarm if the water consumption deviates, and it is a very simple way to detect diarrhoea… It calculates the water consumption based on previous rounds. It uses artificial intelligence to calculate logarithms for what the consumption will look like in the future.” Speaker 5, pig focus group 2.

“We can also get an alarm on sows that have a poor appetite, that do not eat fast enough and do not eat their entire daily ration. If we see that a sow does not eat up, and has left a kilo for three days, they will appear on a list where we need to check.” Speaker 1, pig focus group 2.

Farmers used individualisation techniques to aid this data collection at the individual animal level. Calves were often housed in solitary pens in the first few weeks of life, which meant that farmers could determine how much each calf was eating or drinking. Some of the cattle farmers stressed the importance of the data collected at this stage of the calves life.

“We have solitary pens during the entire milking period and have a very low calf mortality. … All calves that get colostrum have a green bucket and those we start to wean and are getting water, gets a blue bucket… since it’s very easy and everyone can do it and that we have the calves in solitary pens.” Speaker 1, dairy focus group 2.

“We have a form where you write when the calf is born, if it (the calving) has gone well and then how much colostrum it drank at the first meal and what (Brix) value the milk had. The intention is to follow up, if there are problems later on, then you can go back and see whether there were any problems in the beginning.” Speaker 4, dairy focus group 2.

“Our routines are such that they are kept in solitary pens for maybe two weeks or something like that, when they have their own individual bucket.” Speaker 2, dairy focus group 3.

Farmers viewed that technologies provided a more objective view of the animals’ bodies. Bos et al. (38) use the notion of “quantified animal” to describe the objectivation of farm animals by precision livestock farming. The objectification of animals has often been approached in studies of the ethics of human-animal relations. Analysis of automatic milking systems on dairy farms shows that there is a three-way ethical relationship between humans, animals, and robotic technology (43). Thus, farm technologies are not ethically neutral. One of the ways in which automatic milking systems (AMS) are positively framed is that they increase the freedom of dairy cows because cows can choose when to be milked (43, 90). Although this can lead to ethical issues when cows resist the AMS (43). Unlike the AMS, the technologies that were used for pigs and youngstock do not change the behaviours of the animals in a way that increases freedom. Instead, the daily activities (e.g., drinking, eating, roaming) of the youngstock and pigs which would otherwise be assessed qualitatively becomes quantified and standardised. The animals are managed by the farmers without the full awareness of the animals because the farmers do not have to be present. The Swedish dairy and pig farmers that used technology in our study justified monitoring their animals with technology because it can improve the animals’ health and welfare. From a Foucauldian perspective, the farmers’ purpose for governing their animals is “the welfare of the population and the improvement of its condition” and use technologies as techniques of government to indirectly impact their animal populations (53). For example, machines can be used which alters the amount of feed given to pigs based on the data it gathers on their feeding history.

“Yes, we have the automatic urban feeder (automatic milk feeder) which is a small computer you can access several times a day and see if they (calves) have drunk as they should… There are a bundle of things you can access and check (on the computer), where there is an alarm.” Speaker 4, dairy focus group 3.

“There is a station where they enter to get their concentrates… they enter, and then it reads if that particular sow should have more, and then it starts to portion out slowly as the sow eats, so that you know that it is exactly that sow that eats. You can tell from about 300 grams how much she has eaten. Because if she were to leave there, even though she has not eaten her ration, then you know that it is not another sow that eats it, but then you know that it is an animal that should have that feed, because the trough closes again, so that no other sow can enter and eat the rest of the feed.” Speaker 1, pig focus group 2.

Although farmers can understand individual animals through their senses and experiences towards the animal body, technologies can individualise animals in a different way. The data collected by technologies can be used to generate individual animal profiles. The quotes below show how these farmers aspire to have technologies that provide “holistic” data about their animals. The animals do not just have their own body, but also hold a datafied body. The body-technology hybrid is referred to as a “cyborg” (36). The presence of this datafied body leads farmers to experience the animal bodies differently. They incorporate this datafied body in their decisions about the animal. This means that animal bodies both shapes, and are shaped by, data.

“I want a more holistic perspective, for each animal. …I don’t just want the number of ruminating minutes or the milk temp. I want to be able to see other things also. To see if there is a problem because if I only get 39.8 then I must also check she is in heat perhaps, and if I only get ruminating minutes and nothing else then I don’t know if it’s because she’s moved to another group or because she has calved recently.” Speaker 3, dairy focus group 2.

“I was also thinking about an activity chip in the sows. That registers their behaviour patterns and activities, but also the temperature and things like that. Like eating patterns and so on.” Speaker 6, pig focus group 1.

#### Groups

Farmers also collected information about their animals at the group level. This was more often depicted in the pig farmer focus groups due to the rearing system. In contrast to the small calves who were often housed in individual pens, sows were housed together with their piglets in pens. The farmers usually had a sow card on each pen which was used to record information. Therefore, data relating to the piglets were recorded with the sow, reflecting a family group. Sows were also placed into batches which reflect birthing groups.

“Yes, we use the current sow card for the current farrowing, we always print that out, so that there is one in paper format with each sow, it hangs in the same chain with the heat lamp, so that it is there. We write everything down there, and then we register it after the farrowing, really all the information.” Speaker 4, pig focus group 2.

“Let’s not even talk about local anaesthesia. We use tons of that. But I just register that “We have castrated more or less this many.” It’s written on the sow’s cards how many males there are and then you just sum it up.” Speaker 1, pig focus group 3.

“I have pigs being born every week. In other words, I have 36 sows farrowing every week, I have 22 groups so I have one group per week.” Speaker 1, pig focus group 3.

The pig farmers also used their pig’s data to decide what groups the pigs should be placed into. Thus, data and technologies facilitated the group-level management. Putting the pigs into sub-groups was a form of biopower as farmers sought to contain different classes of pigs for the benefit of the population (91). Farmers try to ensure that their sows are in homogenous groups to facilitate the reproduction management process in the all-in/all-out system. For example, Speaker 5 grouped sows by weight and fat. Those that were below (or above) the correct weight were placed together in order to correct their deviation from the norm.

“I group them according to fat and weight, you could say, and also age. It is quite a lot of work to get it together, but our sows are more equal, and they keep the correct fat more now, going into the farrowing stable, and that has been the priority.” Speaker 5, pig focus group 2.

Consequently, technologies were often used at a group level for pigs. One example of a group-level technology used on pig farms were group weighing scales:

“There is a scale in the floor. If you enter the growth section, there are four stables on one side and four on the other. Then there is a corridor. Before the stables begin in the corridor, there is a scale. All the width of the corridor… You grab a litter from the birthing centre, walk with them to the balance, note the number on the list, and then WinPig calculates the average.” Speaker 6, pig focus group 1.

Another example was an early warning system which used an algorithm to detect group-level deviations in drinking behaviour:

“Speaker 4: There have been a few times when we have detected post-weaning diarrhoea in groups, and then you then get an alarm that the water consumption is deviating.

Speaker 2: Is it located in each individual water nipple?

Speaker 4: No, where we measure water consumption at a department level. Because if you have a major outbreak of diarrhoea or something, you cannot detect it at pen level, but if you get diarrhoea in a whole section, then it is easier to have a text message or alarm on the phone.” Pig focus group 2.

Therefore, farmers grouped pigs by their biological characteristics. These groups were socially constructed by farmers based on their biomedical knowledge of the pigs, a concept called “biosocial collectivities” (54, 58). Holloway et al. (58) shows the relevance of biosocial collectivities in livestock farming through analysing how sheep and beef breeds are constructed through formation of groups of people and animals. From the perspective of the Swedish pig farmers, the biosocial collectivity included the pigs that have been grouped, and the farmers and workers that manage them. It is the farmer who enforces the groupings on the pigs and therefore the farmers and workers must work on the pigs to improve the health and welfare of population. Our study shows that data recording at the group level facilitates formation of the biosocial collectivities. The technologies described above give farmers new knowledge and generate standards about the performance of pigs with certain biological characteristics. This allows groups of animals to be compared to the herd population. Therefore, the pigs must conform both to group-level and population-level norms. For example, they may have to eat or drink the right amount for their litter, age, or weight group.

Dairy farmers also constructed sub-groups of youngstock based on characteristics of the animals. For example, after the first few weeks in solitary pens, calves were transferred to small group pens. In some cases, these sub-groups were used for group-level data collection (e.g., Speaker 3, dairy focus group 2).

“Our calves stay in a solitary pen for a week. Then they are moved down to the group pen with at most 12 calves in that pen, but we often sell the bull calves by three weeks of age. In the end, there will be seven heifers left, usually for seven to three months.” Speaker 3, dairy focus group 1.

“I have decided that from the time they are moved to the common pen, when they are about ten days old, they should be measured once a week. The largest, a medium and the smallest calf in the group. So that you have on paper that there is a difference. So, what I do is that I write down the group, calf, date, and weight for these three.” Speaker 3, dairy focus group 2.

#### Populations

The farmers also collected data at the population level to understand the overall productivity and health of their herd. The following quotes show how the farmers tried to regulate their farm population by managing sows and cows so that they have appropriate birth rates. Artificial insemination was used as a technique to ensure the correct number of animals were pregnant at the correct time, and data was used to determine and assess these numbers and timings.

“We look at how many (heifers) have calved and how many will calve in the future. We have a goal of how many calves we want per month and then we get a prognosis, also calculated according to the other calvings (by cows) and then we calculate. Now we are planning to inseminate 13–14 animals per week to get 32 calvings a month and then you work on that. We are aiming for more heifers. We want to inseminate cows.” Speaker 2, dairy focus group 2.

“We are part of Gård och Djurhälsan (Farm and Animal Health—the name of a company that provides health services) and the vet sends us different diagrams, when we have sent in all our data, we get the diagrams for next time. We go over them, so we know if there’s a lot of diarrhoea, a lot of arthritis, a lot of foot abscesses amongst the piglets.” Speaker 4, pig focus group 3.

“So (employee) does a lot of follow-ups both in Excel and in WinPig, so we pull out results on each group and of course we also pull-out quarterly reports, etc… we usually inseminate up to approximately 60 sows, because we also sell some purebred in each group. The recruitment is quite high, because we have a breeding herd, so we usually inseminate between 57 and 60 animals, and we want 50 in the group. And there are usually about four who are not pregnant each time. So, if we have a lot, then we have to analyse.” Speaker 4, pig focus group 2.

In this case we conceptualise the population level as the individual farm, but it also possible for animals to be governed through biopower at more regional or national levels. For example, in Sweden the farmers are not allowed to treat their animals without a consultation with the veterinarian. Some of the dairy farms had ViLA (delegated drug use) contracts, which means that a specific veterinarian and a specific farmer have a contract between each other defining for which disease symptoms the farmer is allowed to initiate treatment without consulting the veterinarian. The farmers must register all medicine use and have regular follow ups with their veterinarian and if their antibiotic use or herd health is judged to be not appropriate, then the farmer will lose the ViLA contract. The farmers and their animals are subjected to biopower by comparing their farm against the national population of farms.

“We have the ViLA status checks every week …We work a lot with preventive animal health care. Partly because we have such close contacts with him (vet) and we follow up on cell counts after test milking and do fertility reviews and such.” Speaker 2, dairy focus group 2.

It should be noted that although many of the farmers talked about technologies on their farm, there were still several who were manually recording data via pen and paper—particularly on pig farms. Sometimes the farmers entered the data into software at a later time, but some did not. This was because of usability problems with the small choice of software available. Manual recording reduces the capability for surveillance of animals and thus animals may be represented differently.

“We note down everything on paper in the stables, both births, weaning and insemination. Then I add it into WinPig. I should do that once a week, but there is not always time, and then all of a sudden you have a back log, because they give birth every week. But we note it down on paper, and then put it into the computer, and by then, if you are 2–3 weeks behind” Speaker 6, pig focus group 1.

In summary, farmers use data and technology as a technique to manage their animals at the individual, group, and population levels. This is an example of biopower as farmers use data to individualise animals and make them more visible, yet simultaneously they use data to manage populations by intervening with fertility. Furthermore, animals are separated into groups to aid management of the population and correct deviations from norms.

### Managing human labour

This final theme focuses on how the farmers used technology and data to manage their workers. Firstly, farmers could use technology to track their workers’ daily activities on the farm. The examples below show that some farmers can track workers’ locations and assign them tasks hour-by-hour. This detailed information allows farmers to evaluate the workers’ activities with greater scrutiny.

“We have an app that is called Check Proof, where you have routines in the app, and you check them off when they are done. It is because there are so many employees here, then it is easy to see what things are done and not done. Then you could move that routine to the next day. In the app you can also add notifications that are sent to the person responsible for repairs, etc.” Speaker 6, pig focus group 1.

“The idea is that different people are assigned different things. Then you go in and follow your instructions and do them, and you are almost clocked by when you start your routine. I can go in and see that it was started at eight in the morning and it was finished at nine, and then they can start a new work-task.” Speaker 1, dairy focus group 3.

The farmers therefore use technologies as a technique of disciplinary power called hierarchical surveillance. This aims to produce individuality through assigning tasks that render workers distinct from each other (92). Through hierarchical surveillance, farmers can manage the activities and distribution of workers across their farm. Such technologies can make farm workers more accountable for their actions.

“I have five employees today, and all people work differently, and it is important to just try to get everyone up and running. And the fact that it is not possible to forget, and it is not possible to put the blame on someone else and say that they were the ones that were supposed to do a certain task.” Speaker 1, dairy focus group 3.

Second, farmers could use technologies or data to increase standardisation of work on the farm. Many of the farmers discussed how they have developed routines and standards which workers have to adhere to. The workers’ knowledge of the animals must be shared with the farmer and other workers on the farm. This was so that workers on the farm could easily be substituted for another. Therefore, farmers were not reliant on one or two key workers. This may be apparent because of the state of the labour force in Sweden. Finding workers with stockperson skills is a major challenge for Swedish farmers (9). As the rural population declines, Swedish farmers often use immigrant labour and contract work which may produce instability in the workforce as staff stay for short periods of time (93, 94).

“So, we have to write it down and my biggest thing that I tell the employees is that everyone has to do it the same way, we can’t be like, “No, I’ll do it like this.” No, that doesn’t work because I should be able to jump in at all positions. I don’t have a position, I just wander around, or I’ll jump in where I’m needed. That means that no matter who have been working, I have to be able to understand what they have written.” Speaker 1, pig focus group 3.

“It’s nice to know, that if I get a stomach flu, that I can stay in bed and anyone can take care of the calves, because if it’s a calf that doesn’t get up from its solitary pen, then something is wrong. It is very easy, even those who do not usually take care of the calves or if you need to call someone who can come and help out.” Speaker 1, dairy focus group 2.

It is important to note that power is not always repressive and it also “*transverses and produces things, induces pleasure, forms knowledge [and] produces discourses*” (95). The use of technology on farms to increase the standardisation of tasks can be viewed as positive and productive for farm workers. Technologies can facilitate communication and collaboration between farmer and farm staff, and among farm staff, which can make their shifts easier by harmonising routines. Furthermore, standardised ways of communication, such as colour coding, allows people who cannot speak Swedish fluently to work on Swedish farms and communicate with others. Therefore, technologies can produce discourses on farms that would otherwise not be there. Furthermore, technologies may help to form knowledge where language barriers exist.

“If you are to bring technology into it all, it is communication. When the next person comes for the next shift, that they know that this calf has not eaten (in the) the previous shift. That they keep their eyes open, because then, in our case, if it does not eat again, and we do not discover anything, this is of course where we must bring in a vet.” Speaker 2, dairy focus group 3.

“The calves that have not drunk should have a red light flashing on the collar and those that you must keep an eye on they should have a blue light that flashes or something like that.” Speaker 1, dairy focus group 2.

“What we use besides manual checklists is that we have different groups in a chat room, “WhatsApp.” Those who take care of the calves can write there if there is something extra besides what is communicated on the checklist elsewhere.” Speaker 2, dairy focus group 2.

The increased standardisation of farm work has the potential to de-skill the farm worker population. Standardisation may help farmers to employ cheaper, unskilled, workers as a contingency for when they lack skilled farm workers who have an “eye for the animals.” These findings contrast with those discussed by Rotz et al. (96), in which dairy farmers hired higher skilled workers with higher retention rates when they adopted automatic milking systems. These differences may be apparent because an automatic milking system removes some of the repetitive tasks for dairy farm workers, whereas technologies for youngstock and pigs can make tasks more repetitive and standardized. Furthermore, technologies that standardise work may produce a labour force that is divided into low skilled workers that conduct the standardised tasks and high skilled workers that operate the technologies (96, 97).

“It would be great to have technical aids, so you don’t have to be so dependent on having the best keeper in the calf barn all the time. It can also be a person who really wants to be outside during the summer.” Speaker 3, dairy focus group 2.

“I’m always writing down the routines so that everyone can follow them. I was thinking the same thing as (Speaker 1) said, that it’s very important that everyone does it the same way. It’s absolutely forbidden to make up your own stuff, in that case you go through it so that it’s clear and so that everyone has the same information.” Speaker 4, pig focus group 3.

Some farmers suggested that staff may be replaced by robots. The increasing standardisation of work tasks on the farms mean that technologies can be used where only one person is needed to control them. In similarity with previous research (98, 99), reducing labour costs and increasing employee efficiency was a concern for farmers. Reducing the number of staff by replacement with technologies was seen as an opportunity to do this. Technologies may also complement more complex cognitive tasks which usually require human reasoning, such as observation of animals to detect problems (97).

“There is the staff issue as well. An employee costs money and at the same time you cannot manage everything by yourself.” Speaker 2, dairy focus group 1.

“I believe that this is the future; to have fewer staff members who can take care of more, without compromising the animal welfare.” Speaker 4, pig focus group 2.

“The main reason for why I invested in it (robot) is to get more efficiency out of the staff, and there is probably lacking in my leadership as well. I think it's so much fun to work by myself.” Speaker 1, dairy focus group 3.

Farmers also perceived that technologies could provide a better working environment by offering more interesting work. This suggests that technologies may be able to “*incite pleasure*” (95), i.e., they make farm work more enjoyable. Some farmers used technologies to attract employees to their farm. Similarly, Lundström and Lindblom (9) suggested that one of the advantages for adopting automatic milking systems on Swedish farms was that it was easier to employ people.

“Then make their working environment as good as possible so they don’t have to only shovel shit. That’s what people think before they come here, “Now you’re going to work with poop and a fork.” For their sake if would be nice if you could have easy tasks and good tools.” Speaker 1, pig focus group 3.

Finally, the adoption of technologies on the farm can also be shaped by the workers on the farm. Farmers considered the workers skills and attitudes towards technologies when thinking about using technologies. For example, Speaker 6 (pig focus group 1) did not register treatments in computer software because herself and the workers on her farm thought that it took too much time, and it was easier to write on paper. Whereas, on Speaker 4’s (dairy focus group 3) farm, the majority of workers were interested in technology and apps, and therefore the tools were adopted.

“No technology is easy, and I have to think about my staff, who are born abroad and have different knowledge and I want to collect a lot of data.” Speaker 4, dairy focus group 2.

“Maybe it is in the future. Here no one has had any interest in (software) so far, but maybe that can be changed.” Speaker 6, pig focus group 1.

“We are very many employees here and everyone except one is interested in doing all this that you ask them to do, with the technology and apps. It has become a clear simplification being able to access an app and check and register there, and everyone knows how to do it. A simple app.” Speaker 4, dairy focus group 3.

This theme therefore shows how farmers can use surveillance as a technique in which workers were de-individualised because it may be possible to replace them with others (workers or machines). At the same time, workers were individualised by the surveillance which disciplines them and makes workers accountable for their actions (100). Technologies may also be productive and positive for workers, e.g., by allowing communication, removing the language barrier, and making working life more enjoyable.

### Study reflections

The data collection for this study was conducted in Swedish and translated into English for analysis. Due to the translation, some of the nuances within participants’ language may have been lost. We have tried to accommodate this by having the translations reviewed by a second translator and then by the Swedish speaking authors.

The focus groups were conducted online for this study. The online format of the focus groups led to minor disturbances in the dialogue in some focus groups due to sporadic failure in internet connection. However, the technical quality and quality of discussion overall was perceived to be high, probably because all participants had been exposed to a higher number of online meetings in the 2 years prior to our study during the COVID-19 pandemic. Furthermore, the online format may have been less intimidating to those farmers that find it difficult to express themselves in non-familiar environments. Lastly, the online format made it easier to participate in the focus groups compared to an in-person meeting, where at least 1 day should have been allocated per participant to accommodate for travel time due to the large distances and the diversity in the geographic location of Swedish farmers. Hence, the online format facilitated the participation of farmers, achieving the desired diversity and sample size for this study.

As our study aimed to investigate the impacts of technology use on farms, we go into less detail about the management and data collection that was conducted without technologies. We acknowledge that some farmers do not use technology in their management practices, and this could be a topic of investigation for future studies. Another possible avenue of research is into the impacts of technology and data on the governance of farmers and farms by other actors such as regulatory bodies and agritech firms. Although we have not covered this in detail in our study, other authors have discussed this issue (63, 101).

## Conclusion

This qualitative study shows some of the social and ethical implications of using—or not using—data and technologies on Swedish pig and dairy farms. We used a critical digital health lens, which—to the authors’ knowledge—had not been used to investigate livestock technologies before. This allowed us to understand the broader complexities of their implications on farms by simultaneously investigating biopolitical and embodiment perspectives. Therefore, we could identify the impacts of technologies on humans and animals at the individual, group, and population level. We show that the implications are complex and extend across relationships between farmers, farm workers, animals, and the technologies.

From an embodiment perspective, technologies can change and form the identities of farmers, their workers, and the animals. The farmers saw their animals as an extension of their own identity as a good farmer. They used data and technologies as a technique of self-improvement to work on this identity. The farmers valued the human abilities to have an “eye for the animals,” to have affective relations with animals and abductive reasoning. This both supports and contradicts with their desire to have standardised routines for their animals and workers. Workers’ identities may change as technologies make farming more accessible to people with different skillsets and backgrounds. The identities of livestock also change in the presence of technologies. The technologies provide farmers with a data double of their animals which can alter what is considered a healthy, productive animal.

From a Foucauldian perspective, the farmers, their animals, and farm workers are subjected to biopower through conforming to norms. The farmers carried out moral assessments around mortality and morbidity levels on the farm and used data as a self-regulation technique to conform to the moral norms. The farm animals were subjected to biopower through techniques such as collecting data and segregation into groups or individual pens. The ability to quantify animal behaviours using technology generated new norms around animal health and productivity at the individual, group, and population level. Previous research suggested that technologies give farmers greater control over management of their animals (102). We have extended this by illustrating the roles of data and technologies in the disciplinary techniques that farmers use on their animals. Farm workers were subjected to hierarchical surveillance by using technologies to collect data on themselves and the animals they looked after. We also show how techniques of biopower can be productive. For example, technologies that were used for the surveillance of workers can help to facilitate communication and collaboration between people. Furthermore, when problems arise on farms, technology may facilitate farmers in finding a solution.

This study contributes to the literature on the social and ethical implications of technologies on farms by considering the context of pig farming and youngstock rearing. Our study is novel in that it demonstrated that technologies other than the automatic milking system, such as automatic feeders, weighing scales, and sensors that detect drinking behaviour, can impact human-animal relations. In contrast to automatic milking systems, these other technologies did not increase freedom in animal behaviours and instead standardized behaviours on farms. This highlights the importance of investigating the effects of other technologies on the human-animal relationship. We show that data is a central aspect in the ways that farmers manage themselves, their animals, and their workforce, and that technology can facilitate the generation of data.

### Implications for responsible innovation

Our research used a novel ambivalence approach to analysis which allows for a greater understanding of the complexities and contradictions that are apparent in using data and technologies on farms. We show many tensions in our analysis. For example, farm animals’ health and welfare may improve through technology use as problems can be detected before they manifest as diseases, which may be seen as a benefit for the animals. Simultaneously, the animals were subjected to intense scrutiny as their behaviours become quantified and medicalised, which may be seen as a negative. Farmers rejected technologies in favour of their own experiential knowledge yet at the same time used technologies and data to facilitate their knowledge. These tensions and contradictions suggest that farmers cannot be dichotomised into those who are “non-adopters” or opposed to technology and those that are “adopters” or acceptors of technologies (31). There is diversity in the ways that farmers implement technologies and the possible impacts of this (99). Therefore, we need to understand the situated practices in which farmers use (or do not use) technologies in their everyday life.

Dominant discourses around livestock technologies relate to animal health, welfare, and productivity (23, 103). However, the welfare discourses depicted by farmers in our study and others tend to prioritise the physical aspects over the social aspects (104). For example, dairy farmers may prioritise the health of their calves by keeping them in individual pens to prevent disease spread and aid data collection at the individual level, but also this leaves a herd animal in isolation. The social determinants of farm work also need to be considered (98, 105). The ways in which technologies can be used as techniques to govern animals, workers, or the farmers themselves may be specific to cultural and political environments and requires attention in other regions and animal species.

Our research also highlights the importance of affective dimensions of human-animal-technology relations. We show that farmers’ sense of identity emerges from the emotional and bodily unison with their animals and that the animals can be seen as an extension of the farmers’ selfhood. This “*inter-species body pedagogics*” had previously been examined for human relations with companion animals (79) and we have illustrated that it also exists between farmers and their livestock. We highlight that technology and data use is not only shaped by the emotional ties between farmers and their animals, but also that technologies can help to form (or break) emotional and bodily bonds between them. Thus, when developing technologies, we need to consider farmers’ feelings and attachments towards the technologies and livestock, including which senses could be enhanced and which senses could be lost (106).

Whilst previous research has shown tensions and exploitation of labour due to technologies (96), our work is novel because it shows that using technologies for surveillance of people and animals was a technique that both individualizes and de-individualizes employees to aid with their management. Many studies have investigated impacts of farm technology using a human-animal-technology relational approach (102). We suggest that the “human” part needs to be separated into farmers and their workers to reflect the disciplinary techniques that farmers use to manage their workers. At present much research focuses on livestock farmers’ experiences of using technology and very few investigate other users, such as farm workers or veterinarians (107). Within the responsible innovation framework, we need to ensure that we understand the perspectives of multiple user groups, including those that might be least empowered.

The findings from this study will be used to inform the development of technologies to aid management of diseases on Swedish pig and dairy farms. Based on these findings we aim to address potential negative consequences of technologies, such as the generation of negative emotions and how alerts to morbidity or mortality could degrade farmers’ identity. We will also try to enhance positive aspects of technologies such as developing abductive reasoning, creativity, human body-animal body sensory relations, and producing communication and collaboration between farmers and their employees. By including farmers at the initial stages of technology development, anticipating the potential consequences of the technologies, and being responsive to farmers’ concerns, our Living Lab approach enables us to achieve the goals of the responsible innovation framework.

## Data availability statement

The datasets presented in this article are not readily available because participant confidentiality and privacy. Requests to access the datasets should be directed to CD, charlotte.doidge@nottingham.ac.uk.

## Ethics statement

The studies involving human participants were reviewed and approved by ETH Zurich Ethics Committee (application: EK 2021-N-224). The patients/participants provided their written informed consent to participate in this study.

## Author contributions

CD and JK contributed to the conception and design of the study. JF, FD, AO, and GV contributed to data collection. CD analysed the dataset and wrote the first draft of the manuscript. All authors read and approved the final manuscript.

## Funding

This project has received funding from the European Union’s Horizon 2020 Research and Innovation Programme under grant agreement No. 101000494.

## Conflict of interest

The authors declare that the research was conducted in the absence of any commercial or financial relationships that could be construed as a potential conflict of interest.

The reviewer CS declared a shared affiliation with the author JF to the handling editor at the time of review.

## Publisher’s note

All claims expressed in this article are solely those of the authors and do not necessarily represent those of their affiliated organizations, or those of the publisher, the editors and the reviewers. Any product that may be evaluated in this article, or claim that may be made by its manufacturer, is not guaranteed or endorsed by the publisher.

## Footnotes

^1^https://decideproject.eu/
