# Supplementary material for: Social and ethical implications of data and technology use on farms: a qualitative study of Swedish dairy and pig farmers
Source: Front Vet Sci. 2023 Aug 22;10:1171107. doi: 10.3389/fvets.2023.1171107 (PMC10477671; doi:10.3389/fvets.2023.1171107)
Supplement: Supplementary file 1 [file Data_Sheet_1.PDF]

Supplementary Material

Social and ethical implications of data and technology use on farms: A qualitative study of Swedish dairy and pig farmers

Charlotte Doidge, Jenny Frössling , Fernanda C Dórea, Anna Ordell, Gema Vidal, Jasmeet Kaler

*** Correspondence:** Charlotte Doidge: charlotte.doidge@nottingham.ac.uk

1 Focus group discussion guide (English version – cattle example)

<u>Welcome and introductions</u>

Research purpose

- Lead moderator to introduce themselves – a researcher at XXX
- Also introduce the assistant(s) and their role
- The purpose of this session today is to share your opinions and ideas on how technology can help you to identify and control diseases in your herd.
- We are really interested to hear your views on:
    - The ways in which you identify diseases in your cattle;
    - Your experiences in controlling respiratory diseases and scours;
    - The ways you use technology in your day-to-day practices on-farm;
    - How technology could be used in the future to improve identification and control of cattle diseases.
- The feedback you provide today will inform the first stage of the development of a tool.
- In addition to today, you'll also have the opportunity to provide further feedback as the concept of the tool is developed further.

Explanation of the session

- The focus group session is expected to last 1hr 30 minutes
- There are a few things to note:
    - There are no right or wrong answers;
    - You do not have to answer any questions you don't want to – your participation is completely voluntary.
- My role in this discussion is as a moderator. I am here to ask questions, listen and make sure everyone has a chance to talk. I appreciate that there will be lots of interesting things to discuss so I may occasionally interject to bring the discussion back on topic or move onto the next discussion point because of the limited time we have.
- The session will be audio/video recorded to allow the research team to write up the interviews. Everything you say will be reported anonymously.

- Does anyone have any questions before we start?

  Make sure all participants provide informed consent to being audio recorded, and then the moderator should start the recording. Note: there will be a written consent form for each participant to fill in prior to the session.

<u>Introductions</u> (10 mins)

- In some parts of this session, we will be using mentimeter. To make sure everyone knows how to use it before we start, we will begin with a test question: What is your favourite food?
  - To use mentimeter go to menti.com on your phone and type in the following code: XXXX. Then submit your favourite food.
  -
- Please can we go around the room so that everyone can introduce themselves – please tell us your name and about the type of farm you run.
  - Probe on: type of production system, number of cows, their role on the farm

<u>Focus group questions</u>

*Questions with * at the end are probes which are more important*

Imagining an ideal future (20 mins)

Take a moment to think about your ideal future farm and how technology can play a role in cattle health and welfare. Set your imagination free – don't worry about what is possible or not possible in the 'real world'. Using mentimeter, please write down some end-goals you would like to achieve on your farm – with a particular focus on cattle health and welfare. Please go back to menti.com and type in the code at the top of the screen.

- **Please share some of your goals for your future farm.**
- **Why do you think this goal is important to you?**
- **How do you think technology and data could help you to achieve your goals?**

Technology use (15 mins)

Technology could be anything from a farm management software, an automatic milking system, or a thermometer, for example.

- **Thinking about your day-to-day practices on-farm, do you use any technologies to help you? If so, please describe**
  - How does it affect the decisions you make?
  - Do any technologies help with identifying or controlling diseases?*
  - *Note: If farmers do not use any technologies on their farm, please use the following question:* If not, can you think of any technologies that help you in your everyday life outside of farming?
- **Are there available technologies that you think work well on your farm? Why do they work well?**

    - *Note: If farmers cannot think of any technologies, probe about farm management software, automatic calf feeders, weighing scales and automatic milking systems (if dairy).*
    - What technologies wouldn't work well on your farm?
    - How do they fit, or not fit, with your farm routine?

Data use (15 mins)

We are interested in your views on any types of data collected on your farm – it doesn't have to be related to cattle diseases. The data collect could be related to cattle movements, medicine use, or finances, for example. Data refers to any information you record – it doesn't have to be using software.

- **What is your experience of collecting and using data on your farm?**
    - What types of data do you collect on your farm?
    - *Probe on: collecting data related to cattle management and disease*, the environment, production.*
    - How do you collect this data? Do you collect any data without the use of technology?
    - How do you utilise the data you collect?*
    - Who do you share the data with?
    - How do they utilise your data?

- **Some of the factors that you might value about the data you collect are currently presented on the screen. Which of these values are the most important to you? Please go back onto menti.com and rank them in the order of what is most important to you.**
    - *Note: The moderators assistant should present the ranking slide.*
    - Why is [value] most important to you?
    - Is there anything that you value about data that is missing from this list?

Identifying and experiencing respiratory diseases and scours (15 mins)

We would like you to focus on respiratory disease and scours as these are two really significant health problems on beef/dairy farms.

- **What is your experience of these health problems in your cattle?**
    - How often does it occur?
    - Does it happen at a particular time or place?
    - How do you think respiratory diseases/scours impact on (welfare, productivity, time, economic costs), if at all?
- **How do you usually identify these health problems in your cattle?**
    - At what stage do you first tend to notice any problems?
    - Any tools/technology that could help you?*
    - Does this identification usually happen at a certain time or place?
    - Why do you think these methods work well for you?
    - What role does your vet have in disease identification?

- **How has the ways you identify and control these health problems in your cattle changed, if at all?**
  - What made you change your practices?*
  - Why do you think your current practice is better?
  - If there have not been any changes: why do you think your practices have stayed the same?
  - *Note: There is no restriction to timeframe for this question – e.g., farmers can talk about changes that happened last year or twenty years ago*

Wrap up (5 mins)

- **What are you taking away from today's discussion?**
- **Are there any final thoughts that anyone would like to share, before we close today's session?**

Thank you all for your contributions today, we really appreciate the thoughts and opinions that you have shared with us around cattle respiratory disease and scours, data and technology use. We will ensure that you will be provided with a copy of the findings from the focus groups.

We are going to use the information you have shared today to develop some ideas of tools that could be useful for farmers like yourselves. If you would be interested in participating in further work on the data tool, such as evaluating the concepts that we come up with and testing out prototypes, please let me know.
